# Adrenal function after induction therapy for acute lymphoblastic leukemia in children short: adrenal function in ALL

**DOI:** 10.1007/s00431-020-03624-5

**Published:** 2020-03-17

**Authors:** Tiia Loimijoki, Risto Lapatto, Mervi Taskinen

**Affiliations:** New Children’s Hospital, University of Helsinki and Helsinki University Hospital, Stenbackinkatu 9, P.O. Box 347, FIN-00029HUS Helsinki, Finland

**Keywords:** Prednisolone, Adrenal insufficiency, ACTH testing, Acute lymphoblastic leukemia

## Abstract

Prednisolone used in the induction phase of the treatment of acute lymphoblastic leukemia (ALL) may suppress hypothalamic–pituitary–adrenal axis and require hydrocortisone substitution. In this retrospective analysis, we reviewed altogether 371 ACTH stimulation tests of 352 children after a uniform NOPHO (Nordic Society of Pediatric Hematology and Oncology) ALL induction. Both low- and standard-dose ACTH tests were used. Full recovery of adrenal function was defined by both normal basal and stimulated cortisol levels. Sixty-two percent of patients were detected with normal adrenal function in median of 15 days after tapering of prednisolone. Both low basal and stimulated cortisol levels were detected in 13% of patients. The median time to normal adrenal function was 31 days (95% CI 28–34), 24 days (95% CI 18–30), and 12 days (95% CI 10–14) for those with basal cortisol <107, 107–183, and >183 nmol/L at first ACTH testing, respectively. Patients with fluconazole prophylaxis had higher median baseline cortisol levels compared to patients without prophylaxis (207 nmol/L, range 21–839 nmol/L vs. 153 nmol/L, range 22–832 nmol/L, *P* = 0.003).

*Conclusion*: These data can be used to reduce unnecessary substitution or testing, but also to guarantee hydrocortisone substitution for those at risk.

**Table:** 

**What is Known:** •*These data can be used to reduce unnecessary hydrocortisone substitution or ACTH testing.* *•Our data helps to guarantee hydrocortisone substitution for those at risk of adrenal insufficiency.*	
**What is New:** •*Full recovery of adrenal function after ALL induction is detected in 62% of patients already at 15 days after tapering of prednisolone.* •*Both basal and stimulated cortisol testing are required for detection of full adrenal recovery.* *•Recovery time of adrenal function is extended over 3–4 weeks after tapering of prednisolone in patients with low basal cortisol levels (<107 nmol/L) at first testing.*

**Electronic supplementary material:**

The online version of this article (10.1007/s00431-020-03624-5) contains supplementary material, which is available to authorized users.

## Introduction

The treatment results of childhood acute lymphoblastic leukemia (ALL) have improved over the last five decades and shown an event-free survival (EFS) of over 90% in patients with favorable clinical and biological risk factors features [8, 13, 15]. This progress is due to intensive multi-agent chemotherapy, better understanding of the biological features of leukemia [5], and not least to rapid evaluation of response to early induction therapy [2, 8, 16].

Corticosteroids have been a fundamental part of induction therapy since the earliest successes in ALL therapy [9]. Both prednisolone and dexamethasone have been used in the contemporary protocols [8, 15, 16]. Nevertheless, the optimal dosing of corticosteroids, to optimize the ratio of efficacy to adverse effects, some of which are lifelong, has not been established [4].

High-dose glucocorticoid treatment is known to cause suppression of the hypothalamic–pituitary–adrenal (HPA) axis, and this may lead to hypoplasia and even atrophy of the adrenal cortex [1]. Thus, the body’s own glucocorticoid production may be suppressed after a long-lasting or high-dose glucocorticoid treatment causing a life-threatening risk if adequate substitution is not administered.

Adrenal insufficiency is a known consequence of a high-dose glucocorticoid treatment during induction therapy of ALL treatment in children. Earlier studies have shown a varying incidence and duration of adrenal insufficiency as well as a controversy regarding the need of hydrocortisone substitution [10]. The ideal time point of testing of the adrenal function during intensive ALL therapy, interpretation of the results and length of hydrocortisone substitution, need further evaluation. The purpose of this study was to find out the prevalence and duration of adrenal insufficiency in pediatric patients after ALL induction therapy with prednisolone.

## Patients and methods

For this retrospective study, we identified all patients (*N* = 352) treated for acute lymphoblastic leukemia (ALL) in the Children’s Hospital, Helsinki University Hospital from 1992 to 2014. During this time, patients have been treated on three consecutive Nordic protocols for ALL: NOPHO-ALL 92 (January 1992 to October 2001), NOPHO-ALL 2000 (January 2002 to June 2008), and NOPHO-ALL 2008 (July 2008 to February 2016). ALL 92 and ALL 2000 protocols included patients from the age of 1 year up to 14.9 years, and ALL 2008 protocol patients from the age of 1 year up to 17.9 years.

**Fig. 1:**
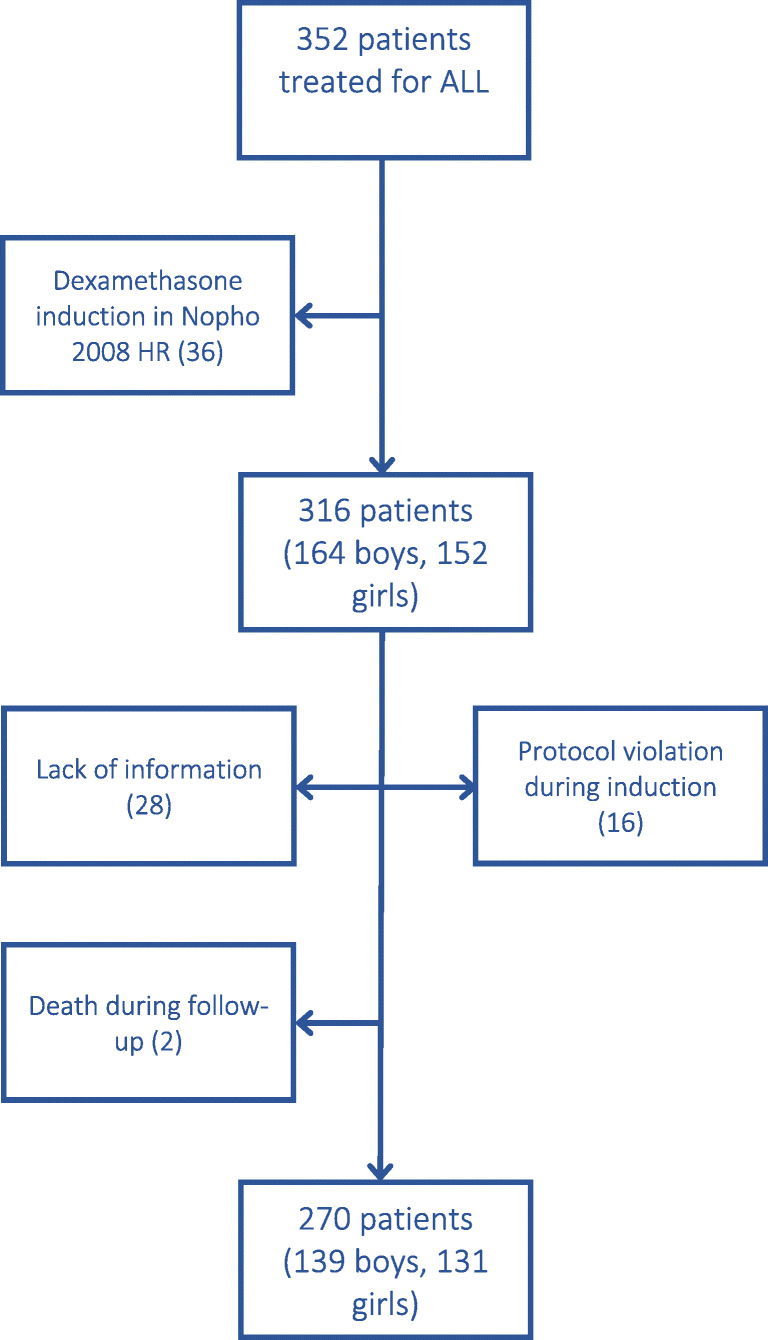
Consort diagram of study patients for analysis of adrenal recovery after prednisolone induction for acute lymphoblastic leukemia. Patients have been treated on NOPHO (Nordic Society of Pediatric Hematology and Oncology) ALL 92 (*N* = 139), ALL 2000 (97), and ALL 2008 (80) protocols

For this study, only patients who had received prednisolone-based induction were included (*N* = 316). This included patients treated with NOPHO-ALL 92 (*n* = 139) and ALL 2000 (*n* = 97) protocols [12], but from NOPHO-ALL 2008 protocol only those with B-cell precursor (BCP) leukemia and white blood cell (WBC) count less than 100 × 10^9^/L at diagnosis [15] (*N* = 80). Altogether, 46 patients were excluded either due to induction failure, missing information, or protocol violation (Fig. 1).

The basic demographic information, leukemia-related parameters, detailed information about the dose and duration of the prednisolone treatment and of the hydrocortisone substitution after induction, information about other medication used during the induction, as well as detailed information about the timing, mode, and results of the ACTH-stimulation tests were collected from the patient records and from the local leukemia registry. Unfortunately, the cytogenetic and minimal residual disease testing was not performed uniformly during the three protocol eras, and, thus, could not be used in the analyses.

The study was approved by the Research Ethics Committee of the Helsinki University Hospital.

### Induction therapy

The induction therapy consisted of prednisolone (60 mg/m^2^/day) for days 1–36 (ALL 92, ALL 2000) or for days 1–29 (ALL 2008), doxorubicin (40 mg/m^2^) two (ALL 2000, ALL 2008) or three (ALL 92) times, vincristine (2 mg/m^2^, capped at 2–2.5 mg) six times (ALL 92 and ALL 2000) or five times (ALL 2008), and intrathecal methotrexate (days 1, 8, 15, and 29). l-Asparginase (*E. coli*) was given intramuscularly at 30,000 E/m^2^/day on days 36–46 (ALL 92) or at 6500 IU/m^2^/day on days 37, 40, 44, and 47 (ALL 2000), or as PEG-asparginase at 1000 U/m^2^ on day 30 (ALL-2008), all given before the first adrenal function tests. Prednisolone was tapered down in 9 days, and hydrocortisone substitution was started the day after (Supplementary Table 1).

### Adrenal function testing

After tapering of prednisolone, hydrocortisone (HC) was given with a recommended dose of 10 mg/m^2^/day until ACTH stimulation test indicated normal HPA function or next treatment phase with corticosteroids was due. This was week 14 in ALL 2008 and ALL 92 and week 28 in ALL 2000 from start of induction.

During the early part of the study period, standard-dose ACTH-stimulation test was performed. In the standard-dose stimulation test, tetracosactide (Synacthen®) was given intravenously at 0.25 mg/1.73 m^2^ in the morning between 7:00 and 9:00. Samples for serum cortisol measurement were drawn before and at 60 and 120 min after the administration of tetracosactide. Due to the change in the institutional policy in 2012, the test was changed to a low-dose test using tetracosactide at 1 μg/1.73 m^2^, and serum cortisol level was measured before and at 30 min after the administration of tetracosactide.

The detection limit of the serum cortisol test (HUSLAB, Helsinki, Finland, immunochemiluminometric assay) was at 20 nmol/L and non-stimulated basal morning values over 69 nmol/L were defined normal. Stimulated cortisol values above 500 nmol/L and 300 nmol/L were considered normal in the standard- and low-dose tests, respectively.

### Statistical analyses

Due to differences in the stratification between the protocols, the National Cancer Institute (NCI) risk classification was used for grouping the patients resulting into three groups: NCI standard risk group (*N* = 164) (B-cell immunophenotype, age <10 years and WBC <50 × 10^9^/L), NCI high risk group (*N* = 85) (B-cell immunophenotype, age >10 years or WBC >50 × 10^9^/L), and T-ALL group (*N* = 21) [14]. Bilineage and biphenotype ALL patients (*N* = 21) were classified into the B cell group.

Mann–Whitney *U* test, Kruskal–Wallis test, and Spearman’s rank correlation tests were used as indicated in the text. These tests were chosen because the material was not normally distributed. *P* values less than 0.05 were considered significant.

Kaplan–Meier and Cox regression analyses were used to create a prognostic model for the recovery of the adrenal function. Normal adrenal function was set as the clinical endpoint. The follow-up time was calculated from the start of HC substitution. Multivariate analyses included only ACTH tests done within 60 days after initiation of HC substitution (the shortest interval to the next protocol phase with corticosteroids). Accordingly, results from 263 ACTH tests were included.

The study population was initially divided into four groups according to the baseline cortisol value (<107.0 nmol/L, 107.01–183.0 nmol/L, 183.01–273.0 nmol/L, <273.0 nmol/L). The purpose of this grouping instead of individual analysis was to have equal-sized groups and more power for the analyses.

The significance of factors included into the univariate analyses were age, gender, risk group, duration until first adrenal testing, use of fluconazole in induction, hydrocortisone dose, cumulative hydrocortisone dose, baseline value of the first adrenal testing, and baseline grouping. A multivariate model for the recovery of the HPA axis was created based on the univariate analysis. A time-dependent Cox regression model was created because the data did not fulfill the proportional hazard assumption required by the standard multivariate Cox regression.

## Results

The final study population included 270 patients with a median age of 4.6 years (range 1.1–16.2). Detailed demographic data of the study population are presented in Table 1. The majority of the patients (95%, 257/270) had no other chronic disease. One patient suffered from diabetes, five were on asthma medication, and seven patients had Down syndrome. Fluconazole prophylaxis was used during induction phase for 158 patients (59%). Only one patient was treated in ICU during the observation period, thus it had no major effect on our results.

**Table 1:** Demographic data on study patients (*N* = 270)

Age at diagnosis in years: mean (range)	4.7 (1.1–16.8)
Gender: girls/boys	131/139
WBC: median (range)	11.8 × 10^9^ (0.5–986.1)
Immunophenotype: BCP-ALL/T cell/MPAL	228/21/21
Risk group: T cell/NCI-SR/NCI-HR	21/164/85

*WBC* white blood cell count, *NCI-SR* NCI standard risk, *NCI-HR* NCI high risk, *MPAL* mixed phenotype acute leukemia

Prednisolone induction therapy lasted for 35 days (median, range 21–60). The exact length of prednisolone therapy varied a little because of clinical reasons, e.g., infections causing minor delays in chemotherapy. Twelve patients had prednisolone therapy over 40 days, all of whom were treated according to ALL 1992 and 2000 protocols (protocol prednisolone duration 36 days). The exact induction phase prednisolone therapy duration was shorter than in the protocol in 83/207 (ALL 92 and ALL 2000) and 36/63 (ALL 2008) patients, and longer in 78/207 (ALL 92 and ALL 2000) and 14/63 (ALL 2008) patients.

Data on 371 ACTH-stimulation tests of these 270 patients were collected. Low-dose and standard-dose ACTH tests were performed to 23 patients (8.5%) and to 247 patients (91.5%), respectively. The first adrenal function test was performed at 10 days (median, range 0–78) from the end of prednisolone course.

**Fig. 2:**
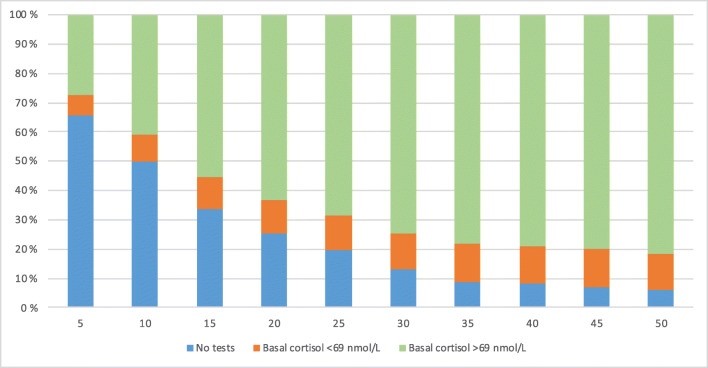
**A**drenal function recovery with time (in days). The figure illustrates the variability in time of testing. It also shows the increasing percentage of patients with normal basal cortisol secretion

The median morning basal cortisol was 184 nmol/L (range, below detection limit–839) in the first test. Altogether, 36 patients (13%) had values lower than the reference range, 10 (3.7% of total study population) of whom had undetectable cortisol levels (Fig. 2). Prepubertal patients (1–9.9 years) showed significantly lower morning basal cortisol levels than older children (10–16.8 years) (*P* = 0.008) (Supplementary Fig. 1).

The median peak result in the first low- and standard-dose ACTH test was 381 nmol/L (range 51–600) and 569 nmol/L (range 29–1853), respectively. In total, 103 patients (38.1%) had an insufficient response in the first test (Table 2), with no difference between low-dose and standard-dose tests (Supplementary Fig. 2). A normal adrenal response was detected at 15 days (median, range 0–78) days after stopping prednisolone. Thirty-four (13.6%) patients had both abnormal basal and stimulated levels. Both a normal basal and a normal stimulated cortisol level were detected in 167 (61.9%) patients (Table 2).

**Table 2:** Basal and stimulated cortisol levels in first ACTH test in 270 patients after ALL induction with prednisolone

	Low dose	Standard dose	Total
Low baseline, low stimulated level	2 (8.7%)	32 (13%)	34 (12.6%)
Low baseline, normal stimulated level	0 (0%)	2 (0.8%)	2 (0.7%)
Normal baseline, low stimulated level	5 (21.7%)	62 (25.1%)	67 (24.8%)
Normal baseline, normal stimulated level	16 (69.6%)	151 (61.1%)	167 (61.9%)
	23	247	270

Basal cortisol levels above 69 nmol/L and stimulated values above 500 nmol/L and 300 nmol/L were considered normal in the standard- and low-dose tests

As expected, patients with insufficient adrenal response in the first test had their test significantly earlier than patients with sufficient adrenal response (median 8 days, range 0–57 days vs. 12 days, range 0–78 days; *P* = 0.025).

Boys tested with the standard-dose ACTH test showed lower stimulated cortisol values than girls in the first test (median 532 nmol/L, range 73–1162 nmol/L vs. 593 nmol/L, range 29–1853 nmol/L; *P* = 0.017). This gender difference was not detected by the low-dose ACTH test (*P* = 0.558).

Patients who received antifungal prophylaxis (fluconazole, a CYP3A4 inhibitor) had significantly higher baseline cortisol levels in the first test than patients without prophylaxis (median 207 nmol/L, range 21–839 nmol/L, two under detection limit vs. median 153 nmol/L, range 22–832 nmol/L, eight under detection limit; *P* = 0.003).

Leukemia-related parameters (immunophenotype, WBC, NCI-risk category) or underlying other diseases did not have an association with the adrenal function results.

### Hydrocortisone substitution

Several patients had multiple ACTH tests before HPA recovery (59 had two, 21 had three or more). Three patients had their test immediately after stopping prednisolone and never received HC substitution.

A sufficient adrenal response was detected in 206/270 patients (76.3%) before the next treatment phase with corticosteroids. A total of 64 patients did not reach sufficient adrenal response before the next treatment phase.

The median hydrocortisone substitution dose was 7.8 mg/m^2^ (range 2.3–34.4). Hydrocortisone substitution lasted for 22 days (median, range 1–120). Interestingly, hydrocortisone substitution was discontinued in 53 patients (19.6%) despite insufficient adrenal response in the stimulation test.

### Factors contributing to full recovery of HPA axis

We defined the full recovery of HPA axis by the patient having both normal baseline and normal stimulated cortisol levels. In particular, we wanted to study the value of basal cortisol level in predicting the duration of HC substitution until the full recovery of adrenal function. To study this, the patients were first divided into four equal-sized groups based on morning basal cortisol values. The two groups with the highest basal cortisol levels showed no difference in the time needed to reach normal adrenal function and were merged into one group including 50% of the patients. Thus, final analysis included three groups: group 1 (*n* = 66) with morning cortisol <107.00 nmol/L, group 2 (*n* = 66) with morning cortisol 107.01–183.00 nmol/L, and group 3 (*n* = 131) with morning cortisol >183.01 nmol/L. These groups are from now on called baseline cortisol value groups. There was no statistically significant difference in time point of the first adrenal test performed (*p* = 0.079) between these groups.

The median time to normal adrenal function in group 1 was 31 days (95% CI 28–34), 24 days in group 2 (95% CI 18–30) and 12 days in group 3 (95% CI 10–14). The probability for recovery changed between days 20 and 30 for group 3, but for the other two groups the recovery was more linear with time (Supplementary Fig. 3). Patients in group 1 (basal cortisol level below 107 nmol/L) have little chance of adrenal function recovery before the next ALL treatment phase with corticosteroids (Supplementary Fig. 3).

We built a Cox univariate regression model to study the patient-, leukemia-, and treatment-related factors on full adrenal recovery: NCI-risk group, cumulative hydrocortisone dose, duration of substitution until first adrenal testing, baseline of the first adrenal test, and baseline groups showed statistically significant impact on recovery. Age, gender, fluconazole use, and HC dose were not statistically significant factors.

**Fig. 3:**
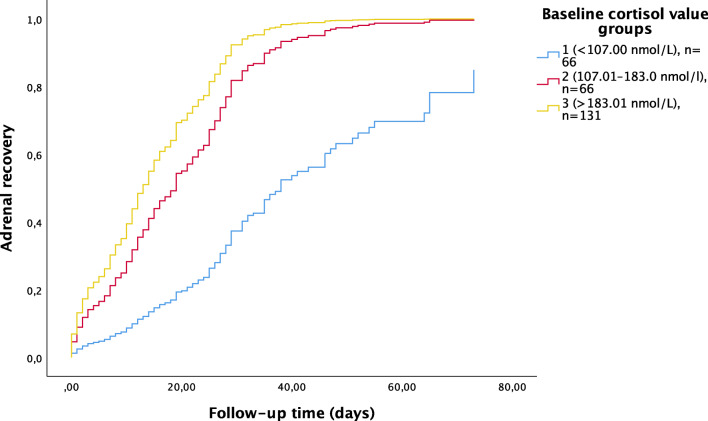
Full adrenal recovery time after prednisolone as function of basal cortisol level. Patients were divided into three groups according to the basal cortisol level at first ACTH test after tapering of prednisolone. Basal cortisol groups <107, 107–183, and >183.01 nmol/L are indicated in blue, red, and yellow, respectively

Then, a multivariate Cox regression model was built with all the variates included in the univariate Cox regression analysis. A model including age, NCI-risk classification, duration until first adrenal test, and baseline group was statistically significant for all variates (Supplementary Table 2). The value of low basal cortisol was even more pronounced in predicting abnormal adrenal function when the variation was adjusted by age, risk classification, and duration until first adrenal testing as contributing factors (Fig. 3).

The proportional hazard assumption was not fulfilled for the duration until the first adrenal test or baseline grouping, meaning that the hazard ratios for these variates are not constant over time. Thus, to control this, we performed a time-dependent Cox regression model starting after day 20 (Supplementary Fig. 3).

Patients in baseline group 2 were 10 times more likely to recover within the observation period (60 days) than patients in group 1, and patients in baseline group 3 were 14 times more likely to recover than patients in group 1. The probability for recovery decreases with age (Supplementary Table 3).

## Discussion

In our study of 270 patients after prednisolone-induction therapy of ALL, many of the patients (61.9%) had a normal adrenal function in stimulation test done 15 days (median, range 0–78) after tapering of prednisolone. Samples were drawn with a wide distribution in time. This shortcoming, however, reflects the clinical real world including varying condition of the patients caused by infections etc.

Adrenal insufficiency defined either as a low baseline or a low stimulated level was detected in 38.1% of patients after 4-week prednisolone induction therapy. This is in line with earlier studies showing that up to 20–70% [3, 7] of children with ALL have adrenal insufficiency after the induction therapy. In addition, Petersen et al. showed that 40% of patients suffer from adrenal insufficiency for over 8 weeks [7]. Cochrane review including 10 studies concluded that impaired adrenal function is common after the induction treatment of childhood ALL and that it usually occurs a few days after tapering of prednisolone [10]. We showed that the earlier the adrenal stimulation test was done, the more likely it showed insufficient response. Our data show that the recovery of the HPA axis is detected in median of 15 days after tapering of prednisolone. This helps in planning of the scheduling of adrenal testing and duration of HC substitution. In addition, we found that morning cortisol values may work well as a screening test for adrenal insufficiency.

Several factors contributed to the recovery of adrenal function. Younger children (<10 years) showed lower morning cortisol values, but the multivariate model indicated that older children are at bigger risk of creating a prolonged adrenal insufficiency. Younger children therefore seem to recover faster. This may be explained by adrenarche after which the relative amount of cortisol production is reduced because of the increased production of sex steroids. No gender difference was detected in the low-dose ACTH test. This may be due to the small sample size (*n* = 23).

Patients who had received antifungal prophylaxis during their induction therapy showed higher morning cortisol values and shorter duration of HC substitution compared to those without antifungals. This is at least partly explained by the inhibition of CYP3A4 by fluconazole. CYP3A4 plays an important role in the metabolism of corticosteroids. The Cochrane review also suggests that clinicians should pay special attention to patients who have received fluconazole during their induction therapy [10]. Earlier studies have shown that fluconazole has a delaying effect on the adrenal recovery with fluconazole doses over 10 mg/kg/day [11]. This may be due to higher steroid concentrations. The prophylactic dosing of fluconazole in our unit has been below this, at 5 mg/kg/day.

The difference between low-dose and standard-dose ATCH-stimulation tests turned out to be smaller than expected. Standard-dose ACTH-stimulation tests are done using supraphysiological ACTH doses which may result in falsely high stimulated cortisol levels. The low-dose ACTH test works as a more physiological simulation of the adrenal function in stress situations. Standard-dose ACTH test has a higher specificity while low-dose is more sensitive but has a lower specificity [6]. Stimulation tests give us more extensive information about the adrenal function than basal cortisol measurements. We analyzed results from two different ACTH stimulation tests. Age was included in the original univariate analyses, but it had no statistical significance.

Twenty-two patients had adrenal function tests misinterpreted resulting in unnecessary hydrocortisone substitution and retesting. Furthermore, hydrocortisone substitution was discontinued in 53 patients (19.6%) despite insufficient adrenal response. These errors point out the difficulty in the interpretation of ACTH test results in clinical setting.

Our study was retrospective, thus the data may have some sources of bias. We had a chance to collect a considerable number of patients and ACTH tests for our analyses. We made an effort to statistically control for the bias caused by age of the patient, timepoints for testing, hydrocortisone dose and duration as well as leukemia-related factors. Multivariate analysis was problematic because of the proportional hazard assumption not being fulfilled. At the end, the results of the time-dependent analysis did not differ much from the traditional Cox regression, but we consider that the time-dependent analyses make the interpretation of the results more reliable considering the usual clinical setting.

Appropriate treatment and testing are required. Uncertainty among clinicians leads to unnecessary testing and incorrect HC substitution endings. The value of morning cortisol indicating sufficient adrenal function varies. We chose the value used by our fully accredited laboratory. The goal of this study was not to set a certain level but to understand the adrenal recovery better. A multi-professional work together with clear instructions improve treatment and reduce error. Based on our study, we recommend weekly morning cortisol measurements after the induction steroid therapy. Once a sufficient level is achieved, HC substitution can be limited to stress situations only, and low-dose ACTH test should be performed. This test should be repeated fortnightly until normal.

## Electronic supplementary material

Supplementary Table 1 (DOCX 26 kb)

Supplementary Table 2 (DOCX 27 kb)

Supplementary Table 3 (DOCX 27 kb)

Supplementary Figure 1 Morning basal cortisol levels according to age. Prepubertal patients (aged 1-9.9 yrs) had significantly lower basal cortisol levels than older children and adolescents (aged 10-16.8 yrs) (PNG 67 kb)

High Resolution (EPS 39 kb)

Supplementary Figure 2 Stimulated corticol levels according to ACTH test used in patients after prednisolone-induction for ALL. Altogether 371 ACTH test are included. Low-dose ACTH test results are indicated in red and the standard dose results in blue. Values above 300 nmol/L and 500 nmol/L were considered normal in the low- and standard-dose tests. (PNG 45 kb)

High Resolution (EPS 61 kb)

Supplementary Figure 3 Kaplan Meier analysis of adrenal recovery time after prednisolone tapering as function of basal cortisol level without adjustments. Patients were divided into three groups according to the basal cortisol level at first ACTH test after tapering of prednisolone. Basal cortisol groups <107 in, 107-183 and >183.01 nmol/L are indicated in blue, red and yellow, respectively. (PNG 71 kb)

High Resolution (EPS 1239 kb)

## Abbreviations

ALL: Acute lymphoblastic leukemia
NOPHO: Nordic Society of Pediatric Hematology and Oncology
