# Supplementary material for: Adrenal function after induction therapy for acute lymphoblastic leukemia in children short: adrenal function in ALL
Source: Eur J Pediatr. 2020 Mar 17;179(9):1453–9. doi: 10.1007/s00431-020-03624-5 (PMC7413907; doi:10.1007/s00431-020-03624-5)
Supplement: Supplementary file 1 — (DOCX 26 kb) [file 431_2020_3624_MOESM1_ESM.docx]

Supplementary Table 1 The Induction therapy of NOPHO-ALL 92, NOPHO-ALL 2000 and NOPHO-ALL 2008 protocols

|  | Prednisolone (60 mg/m^2^/day) | Doxorubicin (40 mg/m^2^) | Vincristine (2 mg/m^2^, capped at 2-2.5mg) | Intrathecal methotrexate | Asparginase |
| --- | --- | --- | --- | --- | --- |
| ALL 92 | Days 1–36 | 3 times | 6 times | Days 1,8,15 and 29 | L-asparginase (E.coli) im. at 30000 E/m^2^/day, days 36-46 |
| ALL 2000 | Days 1–36 | 2 times | 6 times | Days 1,8,15 and 29 | L-asparginase (E.coli) im. at 6500 IU/m^2^/day, days 37, 40, 44 and 47 |
| ALL 2008 | Days 1–29 | 2 times | 5 times | Days 1,8,15 and 29 | PEG-asparginase at 1000 U/m^2^, day 30 |
