# Supplementary material for: Adrenal function after induction therapy for acute lymphoblastic leukemia in children short: adrenal function in ALL
Source: Eur J Pediatr. 2020 Mar 17;179(9):1453–9. doi: 10.1007/s00431-020-03624-5 (PMC7413907; doi:10.1007/s00431-020-03624-5)
Supplement: Supplementary file 2 — (DOCX 27 kb) [file 431_2020_3624_MOESM2_ESM.docx]

Supplementary Table 2. Multivariate Cox-regression analysis of factors contributing to full adrenal recovery after prednisolone-induction for ALL. NCI-SR patients have white cell count (WBC) <50x10^9^/L and age <10 yrs at diagnosis; NCI-HR patients have WBC >50x10^9^/L or age >10 yrs at diagnosis. Baseline groups 1, 2, 3 were formed according to the basal cortisol level at first ACTH test at <107, 107-183 or >183 nmmol/L, respectively.

|  | HR | CI | P |
| --- | --- | --- | --- |
| Age | 0.934 | 0.892–0.978 | 0.003 |
| Risk group |  |  | 0.039 |
| NCI-HR | 0.509 | 0.302–0.858 | 0.011 |
| NCI-SR | 0.638 | 0.386–1.055 | 0.080 |
| Duration until first adrenal testing | 0.945 | 0.933–0.958 | < 0.001 |
| Baseline group |  |  | < 0.001 |
| Group 2 | 3.644 | 2.271–5.848 | < 0.001 |
| Group 3 | 5.499 | 3.665–8.250 | < 0.001 |
