# Supplementary material for: Adrenal function after induction therapy for acute lymphoblastic leukemia in children short: adrenal function in ALL
Source: Eur J Pediatr. 2020 Mar 17;179(9):1453–9. doi: 10.1007/s00431-020-03624-5 (PMC7413907; doi:10.1007/s00431-020-03624-5)
Supplement: Supplementary file 3 — (DOCX 27 kb) [file 431_2020_3624_MOESM3_ESM.docx]

Supplementary Table 3. Multivariate time-dependent Cox-regression analysis of factors contributing to full adrenal recovery after prednisolone-induction for ALL. NCI-SR patients have white cell count (WBC) <50x10^9^/L and age <10 yrs at diagnosis; NCI-HR patients have WBC >50x10^9^/L or age >10 yrs at diagnosis. Baseline groups 1, 2, 3 were formed according to the basal cortisol level at first ACTH test at <107, 107-183 or >183 nmmol/L, respectively.

|  | HR | CI |  |
| --- | --- | --- | --- |
| Age | 0.947 | 0.905–0.991 | 0.020 |
| Risk group |  |  | 0.040 |
| NCI-HR | 0.509 | 0.301–0.860 | 0.012 |
| NCI-SR | 0.641 | 0.385–1.064 | 0.086 |
| Duration until first adrenal testing | 0.883 | 0.861–0.906 | < 0.001 |
| Baseline group |  |  | < 0.001 |
| Group 2 | 10.180 | 4.657–22.253 | < 0.001 |
| Group 3 | 13.713 | 6.771–27.769 | < 0.001 |
