# Supplementary figures and images for: Adrenal function after induction therapy for acute lymphoblastic leukemia in children short: adrenal function in ALL
Source: Eur J Pediatr. 2020 Mar 17;179(9):1453–9. doi: 10.1007/s00431-020-03624-5 (PMC7413907; doi:10.1007/s00431-020-03624-5)

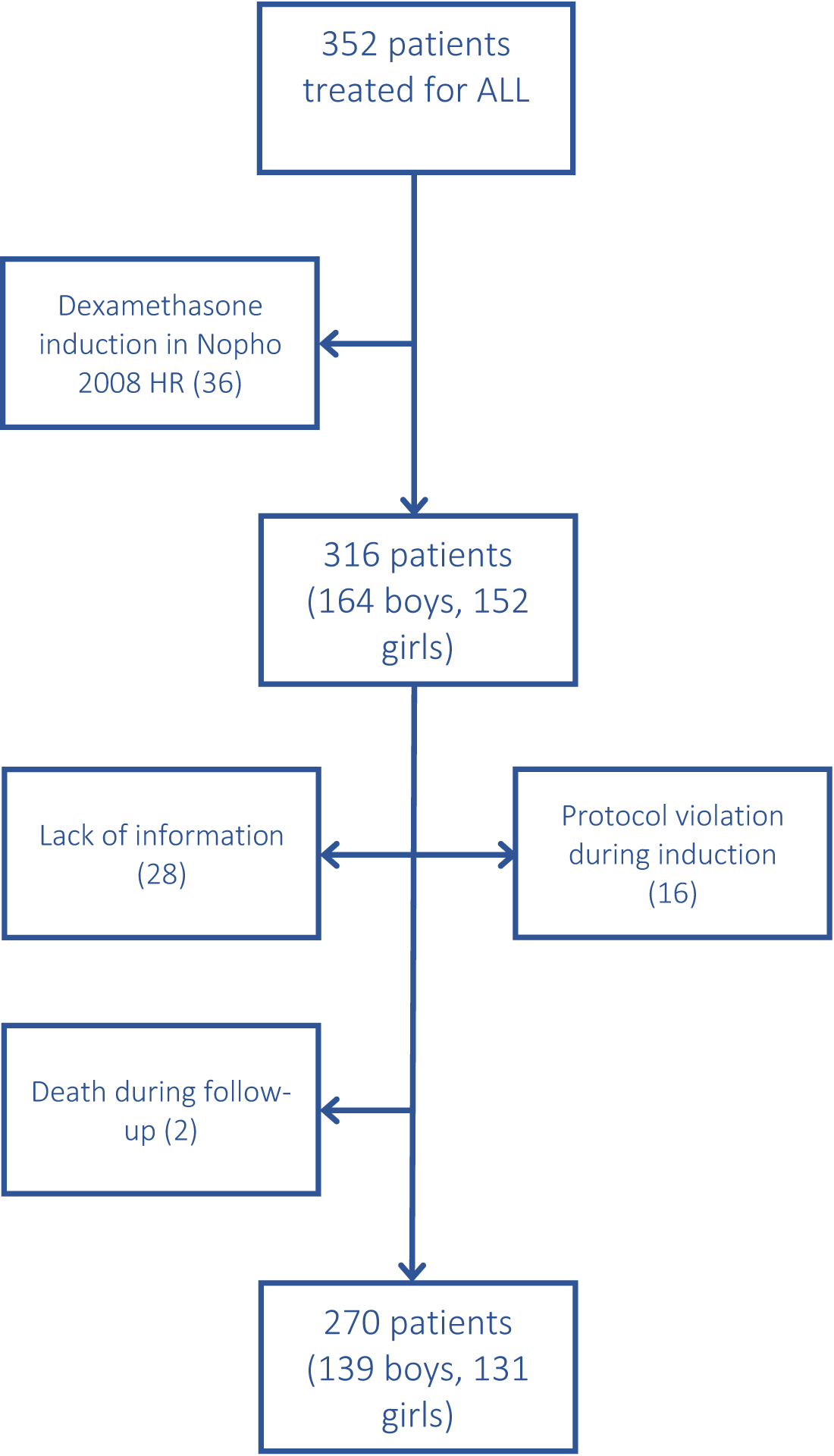

Supplement: Supplementary file 4 — Morning basal cortisol levels according to age. Prepubertal patients (aged 1-9.9 yrs) had significantly lower basal cortisol levels than older children and adolescents (aged 10-16.8 yrs) (PNG 67 kb) [file 431_2020_3624_Fig4_ESM.png]

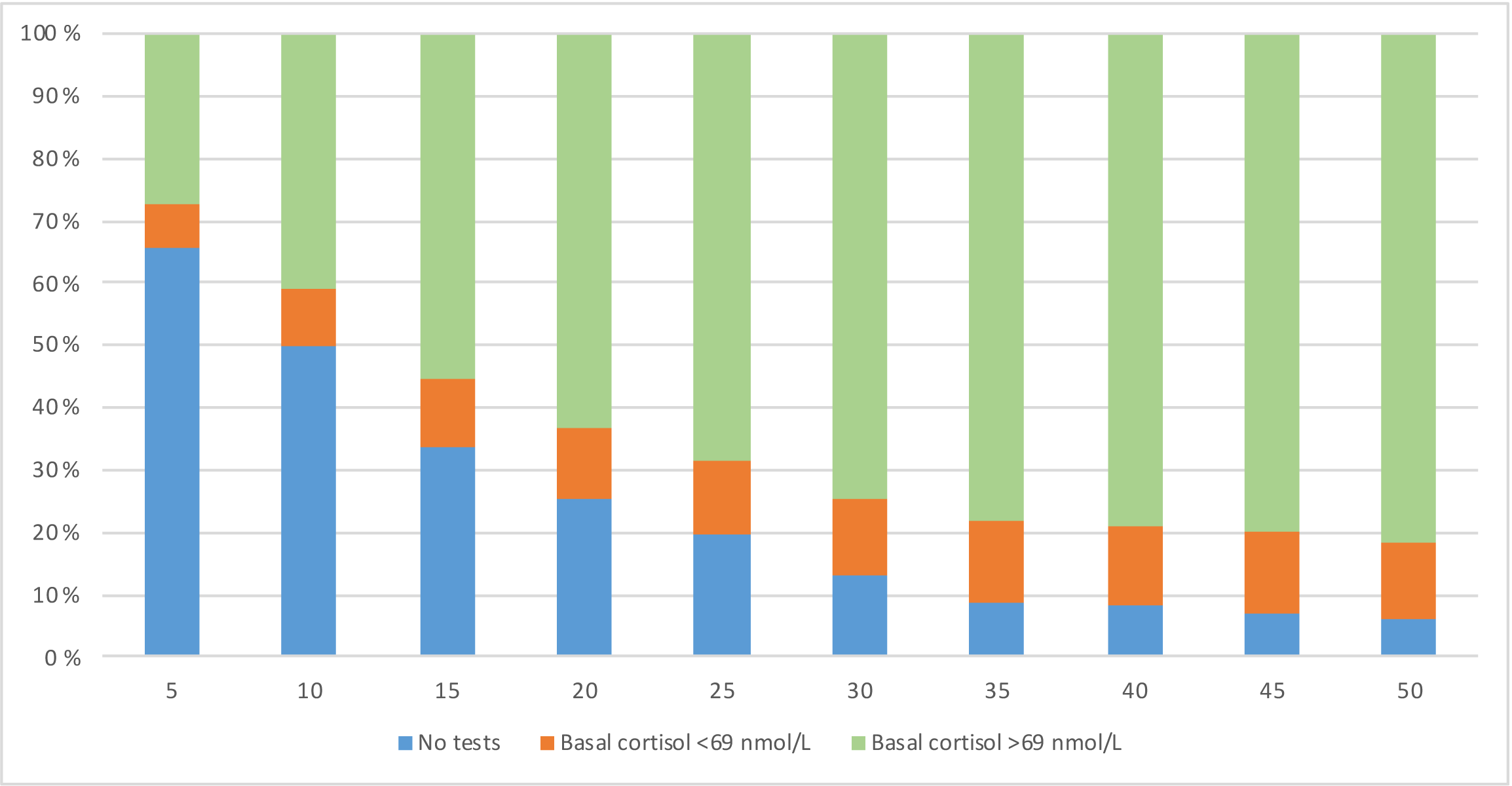

Supplement: Supplementary file 6 — Stimulated corticol levels according to ACTH test used in patients after prednisolone-induction for ALL. Altogether 371 ACTH test are included. Low-dose ACTH test results are indicated in red and the standard dose results in blue. Values above 300 nmol/L and 500 nmol/L were considered normal in the low- and standard-dose tests. (PNG 45 kb) [file 431_2020_3624_Fig5_ESM.png]

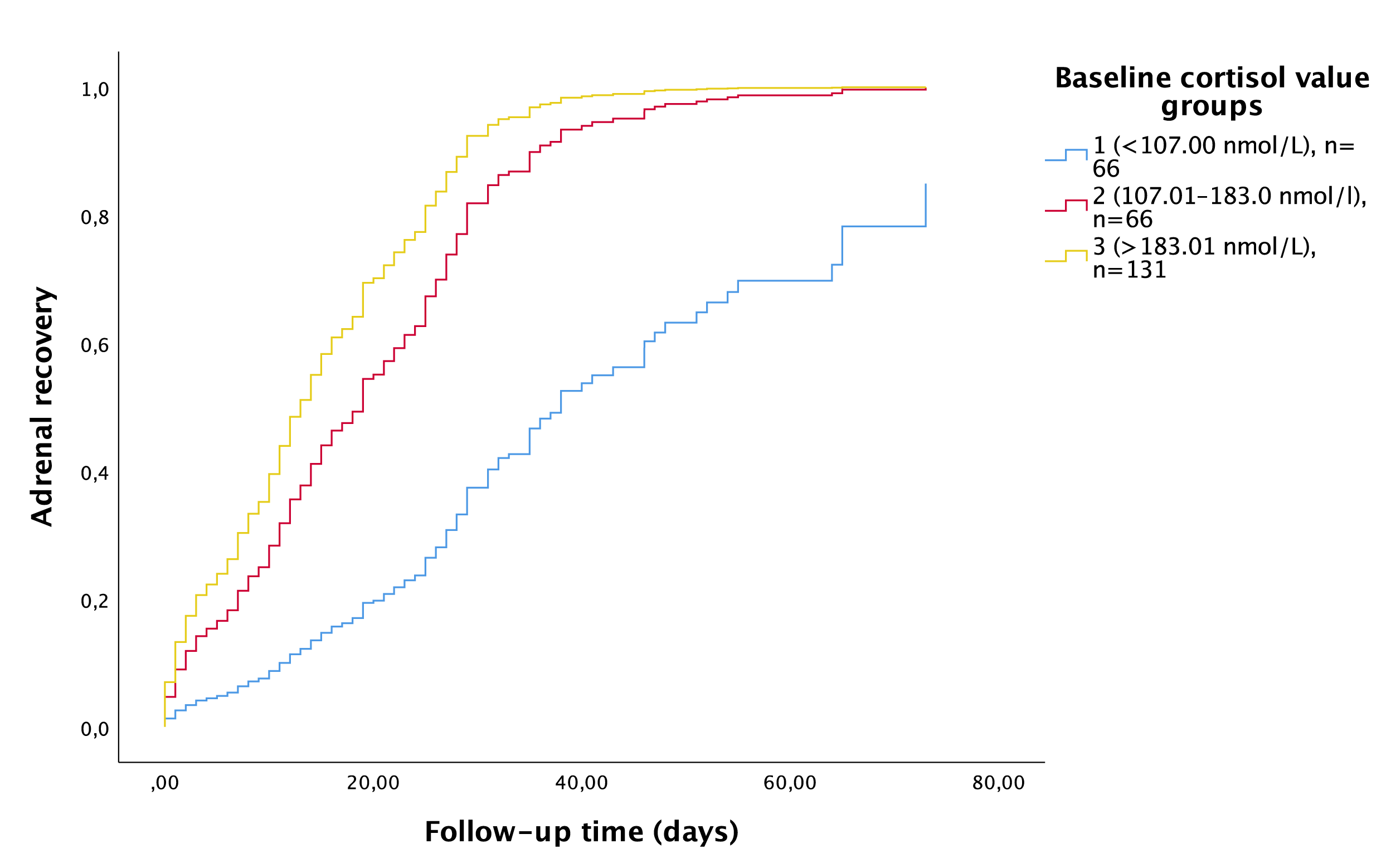

Supplement: Supplementary file 8 — Kaplan Meier analysis of adrenal recovery time after prednisolone tapering as function of basal cortisol level without adjustments. Patients were divided into three groups according to the basal cortisol level at first ACTH test after tapering of prednisolone. Basal cortisol groups <107 in, 107-183 and >183.01 nmol/L are indicated in blue, red and yellow, respectively. (PNG 71 kb) [file 431_2020_3624_Fig6_ESM.png]
